# Effects of Mannan Oligosaccharides on Growth, Antioxidant and Immune Performance, and mTOR Signaling Pathway in Juvenile Tilapia (*Oreochromis niloticus*)

**DOI:** 10.3390/ani15162459

**Published:** 2025-08-21

**Authors:** Qin Zhang, Luoqing Li, Ziyi Ma, Wenyan He, Enhao Huang, Liuqing Meng, Lan Li, Tong Tong, Huizan Yang, Yongqiang Liu, Haijuan Liu

**Affiliations:** 1 Guangxi Key Laboratory for Polysaccharide Materials and Modifications, Guangxi Marine Microbial Resources Industrialization Engineering Technology Research Center, School of Marine Sciences and Biotechnology, Guangxi Minzu University, 158 University Road, Nanning 530008, China; zhangqin@gxmzu.edu.cn (Q.Z.); liluoqing@stu.gxmzu.edu.cn (L.L.); maziyi@stu.gxmzu.edu.cn (Z.M.); hewenyan@stu.gxmzu.edu.cn (W.H.); huangenhao@stu.gxmzu.edu.cn (E.H.); mengliuqing@stu.gxmzu.edu.cn (L.M.); lilan@stu.gxmzu.edu.cn (L.L.); tongtong@gxmzu.edu.cn (T.T.); 2 Guangxi Key Laboratory for Aquatic Genetic Breeding and Healthy Aquaculture, Guangxi Academy of Fishery Sciences, 8 Qingshan Road, Nanning 530021, China; yhzyang@163.com; 3 Guangxi Academy of Marine Sciences, Guangxi Institute of Oceanology Co., Ltd., 92 Changqingdong Road, Beihai 536000, China

**Keywords:** mannan oligosaccharides, serum biochemical parameter, immunity, mTOR signaling pathway, tilapia

## Abstract

Mannan oligosaccharide (MOS), a prebiotic derived from yeast cell walls, has been shown to enhance growth performance and health status in various aquatic species. As an exogenous antigen adjuvant, MOS modulates T-cell-mediated immune responses, thereby improving immune function and suppressing excessive inflammatory reactions. This study aimed to evaluate the effects of dietary MOS supplementation on growth performance, serum biochemical parameters, muscle composition, digestive enzyme activity, antioxidant and immune status, and the mTOR signaling pathway in juvenile GIFT tilapia (*Oreochromis niloticus*). Juveniles (initial body weight: 16.17 ± 1.32 g) were randomly assigned to six treatment groups (three replicate tanks per group) and fed diets supplemented with MOS at 0, 0.2%, 0.4%, 0.6%, 0.8%, and 1% (equivalent to 0, 2, 4, 6, 8, and 10 g/kg of diet, respectively) for 60 days. Compared with the control group, fish fed MOS-supplemented diets exhibited significantly higher (*p* < 0.05) weight gain rates, specific growth rates, and protein efficiency ratios, along with a significantly lower (*p* < 0.05) feed conversion ratio. Serum albumin, high-density lipoprotein, and lysozyme levels were significantly increased (*p* < 0.05), whereas triglycerides, low-density lipoprotein, aspartate aminotransferase, and alanine aminotransferase levels were significantly decreased (*p* < 0.05). In the liver, head kidney, and spleen, the expression of pro-inflammatory genes (tumor necrosis factor α, interleukin 1β, interleukin 6, interleukin 8, and interferon γ) was significantly downregulated (*p* < 0.05), while the expression of antioxidant and protective genes (superoxide dismutase, catalase, glutathione peroxidase, glutathione S-transferase, nuclear factor erythroid 2-related factor 2, lysozyme, alkaline phosphatase, interleukin-10, transforming growth factor β, and heat shock protein 70) as well as mTOR signaling pathway-related genes (mammalian target of rapamycin, akt protein kinase B, phosphatidylinositol 3 kinase, and ribosomal protein S6 kinase polypeptide 1) was significantly upregulated (*p* < 0.05). Overall, MOS positively affects tilapia’s growth, health, and immunity, with 0.60% identified as the optimal dietary level based on growth performance.

## Simple Summary

Mannan oligosaccharide (MOS) has been widely studied for its potential benefits in improving the growth and health status of aquatic animals. This study explored the effects of MOS on juvenile GIFT tilapia (*Oreochromis niloticus*). Juveniles (initial body weight: 16.17 ± 1.32 g) were divided into six groups and fed diets containing 0%, 0.20%, 0.40%, 0.60%, 0.80%, and 1.00% MOS for 60 days. The results showed that MOS-supplemented diets significantly improved growth, serum health indicators, and antioxidant capacity and significantly increased the gene expression of anti-inflammatory factors and the mTOR signaling pathway, while decreasing the gene expression of pro-inflammatory factors. Overall, MOS positively affects tilapia’s growth, health, and immunity, with 0.60% identified as the optimal dietary level based on growth performance.

## 1. Introduction

According to the report of the Food and Agriculture Organization of the United Nations, the genetically improved farmed tilapia (GIFT, *Oreochromis niloticus*) is one of the most extensively cultivated fish species worldwide [1]. It is distinguished by its rapid growth rate, tender flesh, strong disease resistance, and exceptional environmental adaptability, which render it an optimal choice for intensive aquaculture [2]. Intensive farming has been demonstrated to increase the yield per unit water volume. Conversely, high-density farming has been shown to result in the accumulation of metabolic waste and the decomposition of uneaten feed. These phenomena contribute to the eutrophication of water bodies and the escalation of the risk of pathogenic microorganism proliferation [3,4]. To address these issues, antibiotics and chemicals have been utilized extensively. This has resulted in the development of drug resistance in animals, the degradation of water quality, and the bioaccumulation of antibiotics and chemicals in final products. Such use has been associated with an accelerated onset of fish diseases, leading to mass mortality and substantial economic losses [5,6]. In this context, prebiotics, when utilized as green feed additives, have emerged as a subject of significant research interest. These additives aim to address challenges in aquaculture by modulating the intestinal microbiota and enhancing the physiological functions of the host organism [7].

Mannan oligosaccharide (MOS), as a yeast cell wall extract, is among the most prevalent prebiotics in aquaculture, exhibiting potential effects on growth promotion, regulation of intestinal microbiota, and immune activation [8]. Regarding the immune and antioxidant systems of fish, MOS has been demonstrated to elicit a response in the innate immune and antioxidant systems of fish, stimulating pattern recognition receptors, related proteins, and the expression of related immune and antioxidant genes, thereby enhancing the defense and antioxidant abilities of fish against pathogens [9]. Studies have shown that MOS could significantly increase the lysozyme activity, phagocytosis index, and immunoglobulin M levels of fish, thereby enhancing their resistance to bacteria and viruses [10]. For example, under ammonia stress, supplementing feed with 0.2% MOS could improve the growth performance, immunity, antioxidant capacity, and intestinal health of carp (*Cyprinus carpio*) [11]. In terms of growth performance, MOS could indirectly promote fish growth by improving intestinal health and nutrient absorption efficiency. It can also increase digestive enzyme activity, promote nutrient digestion and absorption, and improve protein utilization. For instance, in a study on European sea bass (*Dicentrarchus labrax*), the addition of 1.6 g/kg MOS to the diet resulted in enhanced growth rate and length [12]. In a separate study on Japanese sea cucumbers (*Apostichopus japonicus*), the incorporation of 4–8 g/kg MOS into the feed led to improved growth performance, elevated trypsin and amylase activity, and augmented intestinal microbiota richness and diversity [13]. With regard to the intestinal microbiota, MOS has been demonstrated to regulate the microbial community structure within the intestines of fish. MOS has been demonstrated to possess the capacity to impede the proliferation of deleterious bacteria, including *Vibrio* and *Aeromonas*, while concurrently fostering the growth of beneficial bacteria such as *Enterococcus* and *Enterobacter*. This modification in the microbial community contributes to the maintenance of ecological balance within the intestines, thereby reducing the likelihood of pathogen colonization [14]. The collective findings of these studies suggest that MOS possesses considerable application value and research significance as a functional additive in the field of aquaculture.

The mammalian target of rapamycin (mTOR) signaling pathway plays a crucial role in regulating cell proliferation, differentiation, metabolism, and survival [15]. Additionally, the mTOR signaling pathway interacts with various growth-related factors, metabolic pathways, and signal transduction pathways, forming a complex regulatory network [16]. The mTOR signaling pathway regulates insulin homeostasis, glucose metabolism, and protein synthesis in aquatic animals through upstream akt protein kinase B (AKT) and downstream ribosomal protein S6 kinase polypeptide 1 (S6K1), thereby affecting the growth and survival of aquatic animals [17]. In aquaculture, the mTOR signaling pathway can be regulated by adding prebiotics to feed, thereby improving the growth and immune capacity of aquatic animals. For instance, research on the crucian carp (*Carassius auratus*) has revealed that feeding them wood oligosaccharides can enhance their intestinal microbiota, phosphatidylinositol 3 kinase (PI3K), and AKT, thereby optimizing the mTOR pathway [18]. Similarly, a study on the white shrimp (*Litopenaeus vannamei*) has demonstrated that feeding them red algae polysaccharide extracts can increase the expression of mRNA related to the mTOR signaling pathway [19]. Collectively, these studies indicate that prebiotics play an important role in regulating the mTOR pathway in aquaculture. This has significant application and research value for cell growth, protein synthesis, and metabolism in aquatic animals. However, current research on MOS in aquatic animals remains fragmented: while some studies have explored its effects on growth or immunity individually, comprehensive investigations integrating its impacts on growth performance, serum metabolism, antioxidant capacity, immune regulation, and underlying signaling pathways (such as mTOR)—particularly their interconnected mechanisms—are scarce, especially in juvenile GIFT tilapia.

The objective of this study was to comprehensively assess the impact of incorporating MOS in feed on the serum biochemical parameters, muscle composition, digestive enzyme activity, antioxidant and immune performance, and mTOR signaling pathway of juvenile GIFT tilapia. The findings of this study will not only address the existing knowledge gap regarding the utilization of MOS in tilapia feed but also provide a substantial theoretical foundation for further research and application of MOS.

## 2. Materials and Methods

### 2.1. Experimental Diets

The MOS (50% active mannan-oligosaccharides) used in this experiment was provided by China Angel Yeast Co., Ltd. (Shanghai, China), and the basal feed was a commercial juvenile tilapia diet produced by Nanning Tongwei Feed Company (Nanning, China). Previous studies have reported optimal dietary MOS inclusion levels of 0.40% for zebrafish (*Danio rerio*) [20], 0.30% for Pacific white shrimp (*Litopenaeus vannamei*) [21], and 1.00% for climbing perch (*Anabas testudineus*) [22]. Prior to the aquaculture trial, 100 juvenile tilapia were selected and fed small batches of experimental diets containing different MOS concentrations to verify that MOS supplementation did not induce stress responses. Based on the literature and pre-feeding observations, the experimental diets were formulated to contain 0%, 0.20%, 0.40%, 0.60%, 0.80%, and 1.00% MOS. All of the diets were ground into a 60-mesh powder using a hammer mill, after which MOS was incorporated into the basal diet at proportions of 0, 2, 4, 6, 8, and 10 g/kg of dry feed. The mixture was thoroughly mixed in a drum mixer for 15 min, and then sterile distilled water (40% by weight) was added to form dough with suitable hardness. The mixture was extruded using a single-screw extruder (Delun, Jinan Delun Machinery Equipment Co., Ltd., Jinan, China) to produce feed pellets with a diameter of 1.0–2.0 mm. The pellets were passed through a 60-mesh sieve to ensure uniform particle size and then dried in an air stream at 30 °C until the moisture content was less than 10%. The dried feed pellets were placed in sealed bags and stored at −20 °C until use. The composition of the experimental diets for juvenile tilapia (g/kg of dried feed) is presented in Table 1.

### 2.2. Experimental Fish and Culture

The 1000 juvenile GIFT tilapia used in the experiment were purchased from the Guangxi Angui Aquaculture Company in Nanning, China. The juvenile GIFT tilapia used in this experiment were approved by the Biomedical Ethics Committee of Guangxi Minzu University, Nanning, China (Approval No. GXMZU-2022-008). The juveniles were acclimatized for 14 days before the formal experiment began. During the acclimation period, the breeding tank utilized recirculating water with a temperature maintained at 26 ± 1 °C, a pH range of 7.0 to 8.0, dissolved oxygen levels kept at or above 8 mg/L, total ammonia nitrogen maintained below 0.3 mg/L, and nitrite nitrogen maintained below 0.1 mg/L, under a 12 h light: 12 h dark artificial photoperiod regime. The juveniles were fed three times daily (09:00, 14:00, and 19:00) with the control diet. Before each morning’s feeding, feces were cleared from the breeding system, and approximately one-third of the total water volume was renewed.

After the acclimation period, 540 juvenile GIFT tilapia were uniform in size, color, and appearance and free of abnormalities, and they were randomly divided into six groups. Each group had three replicates, for a total of 18 aquariums. The initial average weight of the fish was 16.17 ± 1.32 g, with a coefficient of variation (CV) of 8.16%. Each aquarium contained 30 juvenile GIFT tilapia and had a capacity of 720 L (150 cm × 60 cm × 80 cm, L × W × H). During the formal rearing experiment, the water quality conditions and feeding schedule were the same as those in the acclimation period. The control group received standard feed without MOS supplementation, whereas the experimental groups were administered feeds supplemented with 0.20%, 0.40%, 0.60%, 0.80%, and 1.00% MOS, respectively. The daily feed intake was set at 5% of the total wet weight of the fish. Each week, three fish from each group were randomly sampled and weighed to calculate the average body weight for adjusting the feed quantity. The feeding cycle lasted for 60 days.

### 2.3. Fish Sampling

After the 60-day experimental period, the juvenile GIFT tilapia were deprived of food for 24 h. From each experimental group, 27 fish (9 fish from each tank) were randomly selected and anesthetized with 0.2 g/L ethyl aminobenzoate mesylate (MS-222; Shanghai Adamas Reagent Co., Ltd., Shanghai, China). The final body weight was measured using a precision analytical balance (Sartorius CPA225D; measurement accuracy ±0.01 g). Blood was collected from the caudal vein and transferred into sterile centrifuge tubes. The samples were stored at 4 °C for 24 h and then centrifuged at 4 °C and 3000× *g* for 15 min. The resulting serum was transferred into labeled centrifuge tubes. Tissue samples, including the liver, muscle, spleen, head kidney, and foregut, were collected separately, placed in pre-labeled sample bags, and immediately stored in liquid nitrogen. All samples were individually packaged and analyzed. Upon completion of all sampling procedures, all specimens were transferred to an ultralow-temperature freezer (–80 °C) for long-term storage and subsequent analyses, including biochemical and gene expression analyses, which were conducted with biological replicates (*n* = 3).

### 2.4. Growth Performance

The weight gain rate (WGR, %), specific growth rate (SGR, %∙day^−1^), feed conversion ratio (FCR), and protein efficiency ratio (PER, %) of the juvenile GIFT tilapia were calculated according to the following formulae, with protein intake for PER estimation determined from feed consumption and the analyzed crude protein content of the diet.

WGR (%) = 100 × [final body weight (g) − initial body weight (g)]/initial body weight (g)(1)

SGR (% · day^−1^) = 100 × [ln(final body weight) − ln(initial body weight)]/days; (2)

FCR = feed consumed (g, dry weight)/[final body weight (g) − initial body weight (g)](3)

PER (%) = weight gain (g)/protein intake (g)(4)

### 2.5. Serum Biochemical Parameter Determination

According to the method of Liu et al. [23], serum biochemical parameters were determined using an enzyme-linked immunosorbent assay (ELISA) analyzer (RT-6100, Rayto, Shenzhen, China) and kits produced by the Nanjing Jiancheng Biotechnology Research Institute (Nanjing, China). The total protein (TP) content in the serum of juvenile tilapia was determined using a protein quantification assay kit (A045-2). Albumin (ALB) content was determined using an albumin test kit (A028-2-1). Triglyceride (TG) content was determined using a triglyceride test kit (A110-1-1). High-density lipoprotein (HDL) was measured using the High-Density Lipoprotein Cholesterol Test Kit (A113-1-1). Low-density lipoprotein (LDL) was measured using the Low-Density Lipoprotein Cholesterol Test Kit (A113-1-1). Globulin (GLB) was measured using the Immunoglobulin G (IgG) Test Kit (H106-1-1). Glucose (GLU) levels were measured using the glucose test kit (A154-1-1). Aspartate aminotransferase (AST) levels were measured using the aspartate aminotransferase test kit (C010-2-1), with one unit of AST activity corresponding to the amount of enzyme that catalyzes the conversion of 1 µmol of substrate per minute at 37 °C. Alanine aminotransferase (ALT) levels were measured using the alanine aminotransferase test kit (C009-2-1), with one unit representing the enzyme’s ability to catalyze the transfer of an amino group from alanine to α-ketoglutarate, producing pyruvate and glutamate, at a rate of 1 µmol per minute at 37 °C. Lactate dehydrogenase (LDH) levels were measured using the Lactate Dehydrogenase Test Kit (A020-2), with one unit of LDH activity corresponding to the amount of enzyme that catalyzes the conversion of 1 µmol of lactate to pyruvate per minute at 37 °C. Lysozyme (LYZ) levels were measured using the Lysozyme Assay Kit (A050-1-1), with one unit corresponding to the amount of enzyme required to lyse bacterial cells and release 1 µmol of reducing sugar per minute. Acid phosphatase (ACP) levels were measured using the Acid Phosphatase Test Kit (A060-2), with ACP activity defined as the amount of enzyme required to hydrolyze 1 µmol of p-nitrophenyl phosphate to p-nitrophenol per minute at 37 °C. Alkaline phosphatase (ALP) levels were measured using the Alkaline Phosphatase Test Kit (A059-2), with one unit of ALP activity corresponding to the enzyme’s ability to catalyze the hydrolysis of 1 µmol of p-nitrophenyl phosphate to p-nitrophenol per minute at pH 10.5 and 37 °C. These tests were performed following the instructions provided in the respective kits.

### 2.6. Muscle Composition Determination

The crude lipid, crude protein, moisture, and ash contents of the muscle tissue of juvenile GIFT tilapia were determined using the methods described by Liu et al. [24]. The crude lipid content was determined by Soxhlet extraction; the crude protein content was determined by the Kjeldahl nitrogen method; the moisture content was determined by drying at 105 °C using the constant weight method; and the ash content was determined by ashing in a muffle furnace at 550 °C.

### 2.7. Digestive Enzyme Activities Determination

According to Zhang et al. [25], the intestine and hepatopancreas of juvenile GIFT tilapia were separately homogenized. The enzymatic activities of lipase, trypsin, and α-amylase were then assessed using the Lipase Assay Kit (A054-2-1), Trypsin Test Kit (A080-2), and α-Amylase Test Kit (C016-1-1), respectively, all obtained from Nanjing Jiancheng Bioengineering Institute (Nanjing, China). Lipase activity was measured based on the enzyme’s ability to catalyze the hydrolysis of triglycerides, with one unit defined as the amount of enzyme that hydrolyzed 1 µmol of substrate per minute. Trypsin activity was determined by the enzyme’s ability to cleave proteins at specific peptide bonds, with one unit defined as the amount of enzyme that catalyzed the hydrolysis of 1 µmol of substrate per minute. The α-Amylase activity was evaluated by its ability to break down starch into reducing sugars, with one unit defined as the amount of enzyme that released 1 µmol of reducing sugar per minute.

### 2.8. Gene Expression Determination

According to the method of Liu et al. [26], Reverse-Transcription Quantitative Polymerase Chain Reaction (RT-qPCR) was performed on samples from the liver, spleen, and head kidney of juvenile GIFT tilapia.

Total RNA was extracted using the Takara MiniBEST Universal RNA Extraction Kit (Universal type), produced by Takara Biomedical Technology (Beijing) Co., Ltd., Beijing, China. Briefly, approximately 100 mg of tissue samples (liver, spleen, head kidney) was ground into a fine powder in liquid nitrogen, followed by the addition of 1 mL of TRIzol reagent and homogenization. The mixture was allowed to stand at room temperature for 5 min. Subsequently, 200 μL of chloroform was added, and the solution was vortexed vigorously for 15 s, followed by incubation at room temperature for 3 min. The sample was then centrifuged at 4 °C and 12,000× *g* for 15 min, and the upper aqueous phase was carefully transferred to a new tube. Next, 500 μL of isopropyl alcohol was added, mixed thoroughly, and incubated at 4 °C for 10 min. The sample was centrifuged again at 4 °C and 12,000× *g* for 10 min, after which the supernatant was discarded. The RNA pellet was washed with 1 mL of 75% ethanol, centrifuged at 4 °C and 7500× *g* for 5 min, and the supernatant was removed. The pellet was air-dried and subsequently dissolved in 50 μL of RNase-free water.

Following the extraction of total RNA from liver, spleen, and head kidney tissues, RNA purity and integrity were evaluated using the following procedures: The purity of RNA was determined by measuring the A260/A280 and A260/A230 ratios, using a NanoDrop 2000 spectrophotometer (Thermo Fisher Scientific, Waltham, MA, USA). In this study, all RNA samples exhibited A260/A280 ratios ranging from 1.8 to 2.0 and A260/A230 ratios of ≥2.0, indicating that the RNA purity fulfilled the required standards for downstream experimentation. RNA integrity was assessed via 1.5% agarose gel electrophoresis in the presence of ethidium bromide. Distinct 28S and 18S rRNA bands were observed, with the 28S band exhibiting approximately twice the intensity of the 18S band, and no signs of RNA degradation were detected. Additionally, RNA integrity was quantitatively evaluated using the Agilent 2100 Bioanalyzer (Agilent Technologies, Santa Clara, CA, USA), yielding RNA Integrity Number (RIN) values of ≥7.5 for all samples, further confirming that the RNA integrity was suitable for subsequent analyses.

The cDNA synthesis was carried out using the PrimeScript™ RT Reagent Kit with gDNA Eraser (TaKaRa Biotechnology, Beijing, China). The 20 μL reaction system consisted of 4 μL of 5× PrimeScript Buffer, 1 μL of PrimeScript RT Enzyme Mix I, 1 μL of Oligo dT Primer (50 μM), 1 μL of random 6-mers (100 μM), 8 μL of RNase-Free Water, and 5 μL of total RNA (approximately 1 μg). The reaction conditions were as follows: reverse transcription at 37 °C for 30 min, followed by enzyme inactivation at 85 °C for 5 s. The synthesized cDNA was subsequently stored at −20 °C.

The following designed primers were utilized: glutathione S-transferase (*gst*), superoxide dismutase (*sod*), glutathione peroxidase (*gsh-px*), nuclear factor erythroid 2-related factor 2 (*nrf2*), catalase (*cat*), tumor necrosis factor α (*tnf-α*), interleukin-1 beta (*il-1β*), interleukin-6 (*il-6*), interleukin-8 (*il-8*), interleukin-10 (*il-10*), interferon gamma (*inf-γ*), alkaline phosphatase (*alp*), heat shock protein 70 (*hsp70*), lysozyme (*lyz*), transforming growth factor β (*tgf-β*), mammalian target of rapamycin (*mtor*), phosphatidylinositol 3 kinase (*pi3k*), Akt protein kinase B (*akt*), and ribosomal protein S6 kinase polypeptide 1 (*s6k1*). *β-Actin* was selected as the non-regulatory internal reference gene. The stability of the internal reference gene β-actin was assessed using GeNorm software. The expression coefficient of β-actin across all sample groups was found to be highly consistent, with a coefficient of variation (CV) less than 5% and an M-value below 0.5 (M < 0.5), indicating stable expression and confirming its suitability as an internal reference gene. All of the above primers were designed using the NCBI Primer-BLAST tool based on the target gene sequences, and their synthesis was carried out by Shanghai Sangon Bioengineering Technology Co., Ltd. (Shanghai, China). Detailed information regarding the primers is provided in Table 2.

The qRT-PCR experiments were conducted using the TB Green^®^ Premix Ex Taq™ II (Tli RNaseH Plus) kit produced by TaKaRa Biotechnology Co., Ltd. (Beijing, China), and they were performed on the LongGene Real-Time PCR System (model Q2000B, LongGene Biotechnology Co., Ltd., Hangzhou, China). Following a thorough examination of the melting curves of the primers, it was determined that all of the primers exhibited single peaks. This finding indicated that the primers were specific to the target sequences for amplification and suitable for subsequent experimental analysis. The 20 μL RT-qPCR reaction system consisted of the following components: 10 μL of Premix Ex Taq II, 0.8 μL each of forward and reverse primers (10 μM), 2 μL of cDNA template, and 6.4 μL of RNase-free water. The reaction procedure was as follows: initial pre-denaturation at 95 °C for 30 s; 40 cycles of amplification, including denaturation at 95 °C for 5 s and annealing at 60 °C for 30 s; finally, a melting curve analysis was performed (95 °C for 15 s, 60 °C for 1 min, and 95 °C for 15 s) to confirm amplification specificity. Relative gene expression levels were calculated using the 2^−ΔΔCT^ method [27].

### 2.9. Data Statistics and Analysis

In this experiment, all of the data were analyzed using one-way analysis of variance (ANOVA) in SPSS Statistics 27. The Shapiro–Wilk test was used to assess the normality of the data distribution, and Levene’s test was applied to evaluate the homogeneity of variances. When significant differences were detected, the least significant difference (LSD) test was performed for multiple comparisons. Data are presented as the mean ± standard deviation (SD). Figures were generated using OriginPro 2021. Different superscript letters indicate statistically significant differences at *p* < 0.05.

## 3. Results

### 3.1. Growth Performance

**Figure 1:**
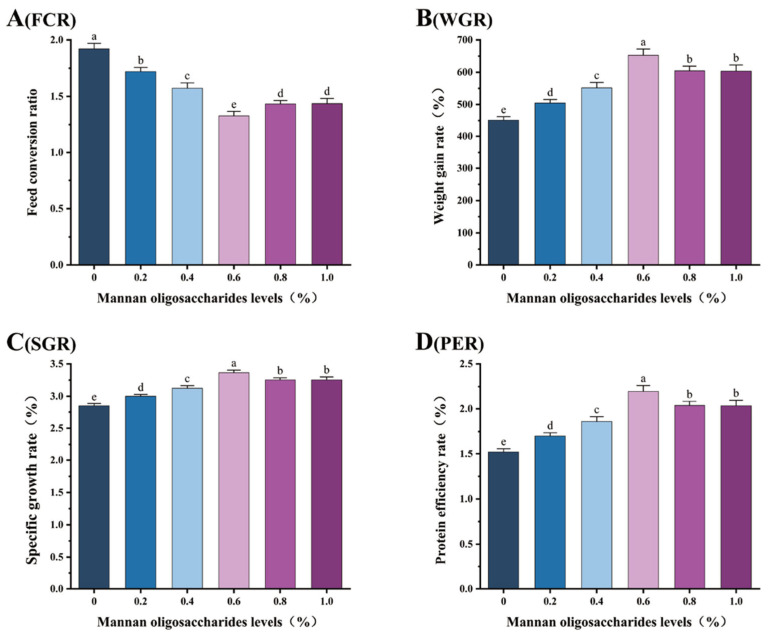
Effects of mannan oligosaccharide supplementation in feed on the feed conversion ratio (FCR, (**A**)), weight gain rate (WGR, (**B**)), specific growth rate (SGR, (**C**)), and protein efficiency ratio (PER, (**D**)) of juvenile GIFT tilapia. All of the above data are the mean ± SD (*n* = 3). Different superscript letters in the figure indicate significant differences among the data (*p* < 0.05).

The WGR, SGR, and PER in juvenile GIFT tilapia fed diets containing 0.20%, 0.40%, 0.60%, 0.80%, and 1.00% MOS were significantly higher than those in the control group (0% MOS) (*p* < 0.05). Among these concentrations, the highest WGR (44.53% improvement), SGR (15.17% improvement), and PER (44.08% improvement) were observed in fish fed 0.60% MOS, and these values were significantly higher than those in fish fed 0.20%, 0.40%, 0.80%, and 1.00% MOS (*p* < 0.05), as shown in Figure 1.

The FCR was significantly lower in all MOS-supplemented groups compared to the control (*p* < 0.05). The lowest FCR value was observed in fish fed 0.60% MOS, showing a 31.15% reduction compared with the control group, and was significantly lower than that in fish fed 0.20%, 0.40%, 0.80%, and 1.00% MOS (*p* < 0.05), as shown in Figure 1.

### 3.2. Serum Biochemical Parameters

The TG, LDL, AST, and ALT contents in the sera of juvenile GIFT tilapia fed diets containing 0.20%, 0.40%, 0.60%, 0.80%, and 1.00% MOS were significantly lower than those in the control group (0% MOS) (*p* < 0.05). Among these, fish fed 0.60% MOS exhibited the lowest TG (decreased by 66.18%), LDL (decreased by 51.32%), and AST (decreased by 14.00%) levels, while the lowest ALT content was observed in fish fed 0.80% MOS (decreased by 12.47%), as shown in Table 3.

The ALB, HDL, and LYZ contents were significantly higher in all MOS-supplemented groups than in the control (*p* < 0.05). The highest HDL and LYZ values were recorded in the 0.60% MOS group, which increased by 129.82% and 83.08%, respectively, whereas the highest ALB content was observed in the 1.00% MOS group, increasing by 57.24% compared with the control.

The TP, GLU, and ALP levels were significantly elevated in fish fed 0.40–1.00% MOS compared with the control (*p* < 0.05). The maximum TP and GLU values were observed in the 1.00% MOS group (increased by 40.21% and 91.10%, respectively), while the highest ALP value was observed in the 0.80% MOS group (increased by 20.07%).

The LDH content was significantly lower in fish fed 0.40–1.00% MOS than in the control (*p* < 0.05), with the lowest value observed in the 0.80% MOS group, representing a 23.56% reduction.

The ACP content was significantly higher in fish fed 0.60–1.00% MOS than in the control (*p* < 0.05), with the highest value occurring in the 0.80% MOS group (increased by 27.58%).

The GLB content did not differ significantly among treatments (*p* > 0.05).

Overall, most of the serum biochemical parameters followed a bell-shaped response pattern, with the greatest improvements observed at 0.60% MOS, after which the effects plateaued or showed a slight decline at higher inclusion levels.

### 3.3. Muscle Composition

The crude protein, ash, and moisture contents in the muscle of juvenile GIFT tilapia fed diets containing 0.20%, 0.40%, 0.60%, 0.80%, and 1.00% MOS were not significantly different from those in the control group (0% MOS) (*p* > 0.05), as shown in Table 4.

The crude lipid content in the muscle of juvenile GIFT tilapia fed 0.60% MOS was significantly higher than that in the control group (*p* < 0.05). Among the experimental groups, no significant difference in crude lipid content was observed in fish fed 0.20%, 0.40%, 0.80%, and 1.00% MOS compared to the control group (*p* > 0.05), as shown in Table 4.

### 3.4. Digestive Enzyme Activity

The activities of lipase, α-amylase, and trypsin in juvenile GIFT tilapia fed diets containing 0.20%, 0.40%, 0.60%, 0.80%, and 1.00% MOS were significantly higher than those in the control group (*p* < 0.05). Among these concentrations, the highest activities of lipase (827.88 ± 19.38 U/mg prot), α-amylase (442.85 ± 9.69 U/mg prot), and trypsin (1747.49 ± 12.52 U/mg prot) were observed in fish fed 0.60% MOS, which were significantly higher than those in fish fed 0.20%, 0.40%, 0.80%, and 1.00% MOS (*p* < 0.05), as shown in Table 5.

### 3.5. Antioxidant Gene Expression

**Figure 2:**
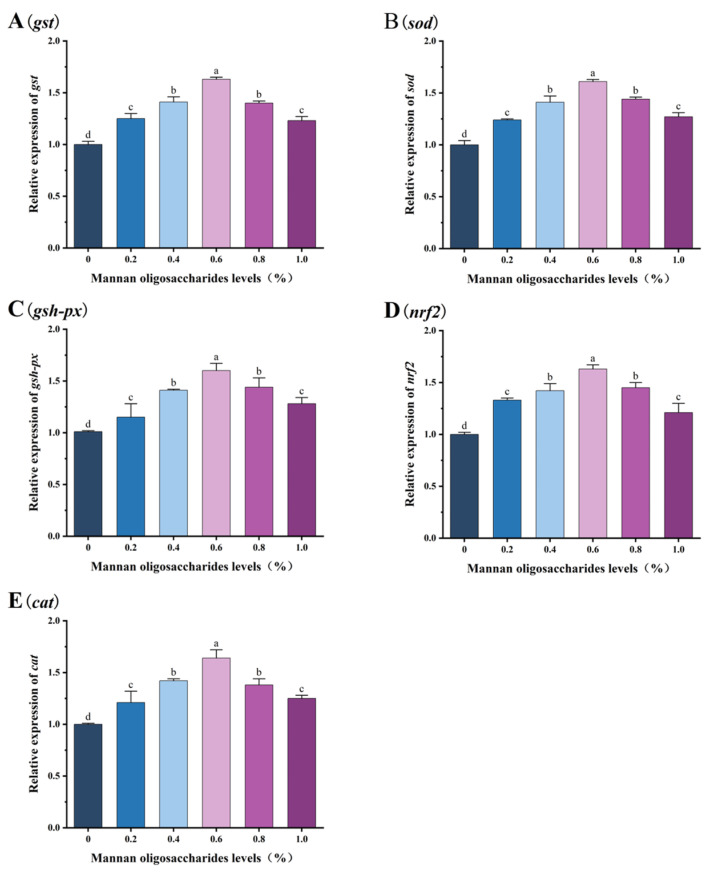
Effects of mannan oligosaccharide supplementation in feed on the relative expression of the glutathione S-transferase (*gst*, (**A**)), superoxide dismutase (*sod*, (**B**)), glutathione peroxidase (*gsh-px*, (**C**)), nuclear factor erythroid 2-related factor 2 (*nrf2*, (**D**)), and catalase (cat, (**E**)) genes in the livers of juvenile GIFT tilapia. All of the above data are the mean ± SD (*n* = 3). Different superscript letters in the figure indicate significant differences among the data (*p* < 0.05).

The relative expression levels of the *sod*, *cat*, *gsh-px*, *gst*, and *nrf2* genes in juvenile GIFT tilapia fed diets containing 0.20%, 0.40%, 0.60%, 0.80%, and 1.00% MOS were significantly higher than those in the control group (*p* < 0.05). Among these concentrations, the highest relative expression levels of the *sod*, *cat*, *gsh-px*, *gst*, and *nrf2* genes were observed in fish fed 0.60% MOS, and these levels were significantly higher than those in fish fed 0.20%, 0.40%, 0.80%, and 1.00% MOS (*p* < 0.05), as shown in Figure 2.

### 3.6. Immune Gene Expression

**Figure 3:**
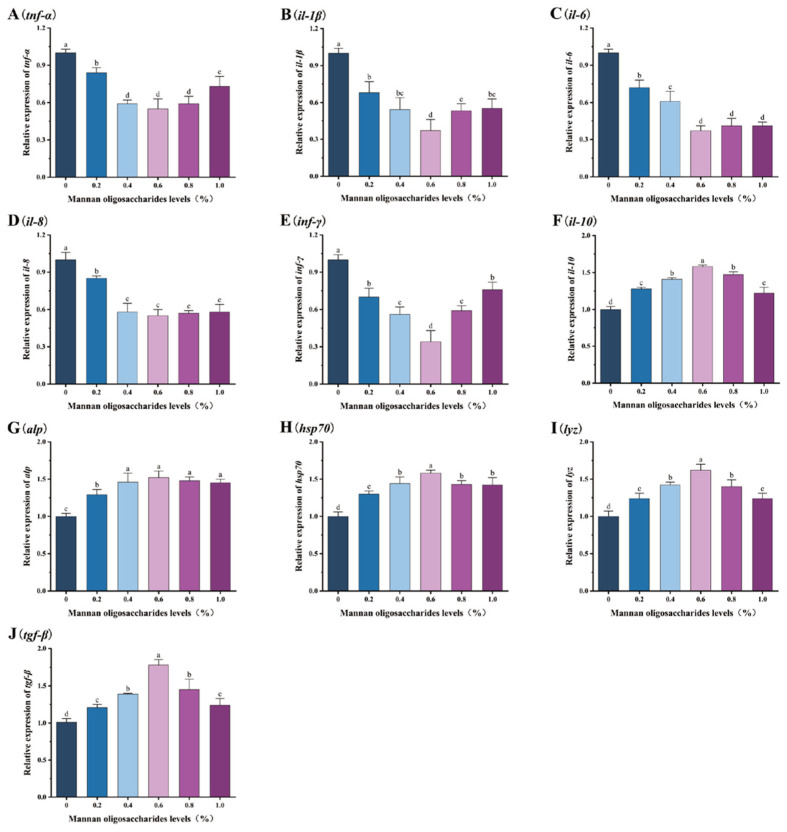
Effects of mannan oligosaccharide supplementation in feed on the relative expression of the tumor necrosis factor α (*tnf-α*, (**A**)), interleukin-1 β (*il-1β*, (**B**)), interleukin-6 (*il-6*, (**C**)), interleukin-8 (*il-8*, (**D**)), interferon gamma (*inf-γ*, (**E**)), interleukin-10 (*il-10*, (**F**)), alkaline phosphatase (*alp*, (**G**)), heat shock protein (*hsp70*, (**H**)), lysozyme (*lyz*, (**I**)), and transforming growth factor β (*tgf-β*, (**J**)) genes in the livers of juvenile GIFT tilapia. All of the above data are the mean ± SD (*n* = 3). Different superscript letters in the figure indicate significant differences among the data (*p* < 0.05).

**Figure 4:**
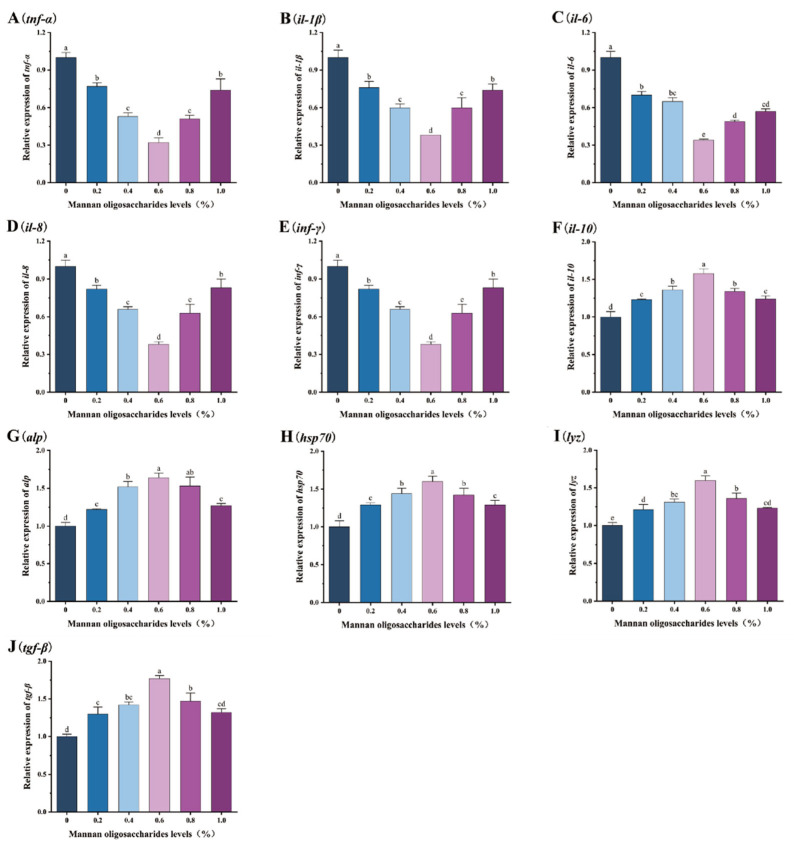
Effects of mannan oligosaccharide supplementation in feed on the relative expression of the tumor necrosis factor α (*tnf-α*, (**A**)), interleukin-1 β (*il-1β*, (**B**)), interleukin-6 (*il-6*, (**C**)), interleukin-8 (*il-8*, (**D**)), interferon gamma (*inf-γ*, (**E**)), interleukin-10 (*il-10*, (**F**)), alkaline phosphatase (*alp*, (**G**)), heat shock protein (*hsp70*, (**H**)), lysozyme (*lyz*, (**I**)), and transforming growth factor β (*tgf-β*, (**J**)) genes in the head kidneys of juvenile GIFT tilapia. All of the above data are the mean ± SD (*n* = 3). Different superscript letters in the figure indicate significant differences among the data (*p* < 0.05).

**Figure 5:**
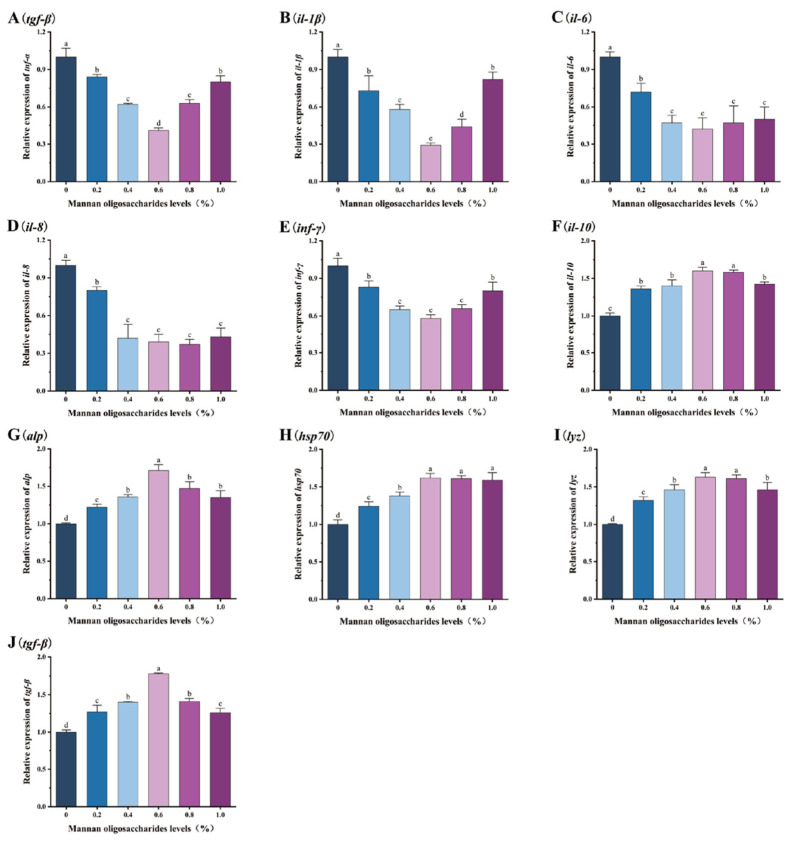
Effects of mannan oligosaccharide supplementation in feed on the relative expression of the tumor necrosis factor α (*tnf-α*, (**A**)), interleukin-1 β (*il-1β*, (**B**)), interleukin-6 (*il-6*, (**C**)), interleukin-8 (*il-8*, (**D**)), interferon gamma (*inf-γ*, (**E**)), interleukin-10 (*il-10*, (**F**)), alkaline phosphatase (*alp*, (**G**)), heat shock protein (*hsp70*, (**H**)), lysozyme (*lyz*, (**I**)), and transforming growth factor β (*tgf-β*, (**J**)) genes in the spleens of juvenile GIFT tilapia. All of the above data are the mean ± SD (*n* = 3). Different superscript letters in the figure indicate significant differences among the data (*p* < 0.05).

The relative expression levels of pro-inflammatory genes (*tnf-α*, *il-1β*, *il-6*, *il-8*, and *inf-γ*) in the liver, head kidney, and spleen of juvenile GIFT tilapia fed diets containing 0.20%, 0.40%, 0.60%, 0.80%, and 1.00% MOS were significantly lower than those in the control group (*p* < 0.05). Among these concentrations, the lowest relative expression levels of the *tnf-α*, *il-1β*, *il-6*, *il-8*, and *inf-γ* genes were observed in fish fed 0.60% MOS, as shown in Figure 3, Figure 4 and Figure 5.

The relative expression levels of anti-inflammatory/protective genes (*il-10*, *alp*, *lyz*, *hsp70*, and *tgf-β*) in the liver, head kidney, and spleen of juvenile GIFT tilapia fed diets containing 0.20%, 0.40%, 0.60%, 0.80%, and 1.00% MOS were significantly higher than those in the control group (*p* < 0.05). Among these concentrations, the highest relative expression levels of the *il-10*, *alp*, *lyz*, *hsp70*, and *tgf-β* genes were observed in fish fed 0.60% MOS, as shown in Figure 3, Figure 4 and Figure 5.

### 3.7. mTOR Pathway Gene Expression

**Figure 6:**
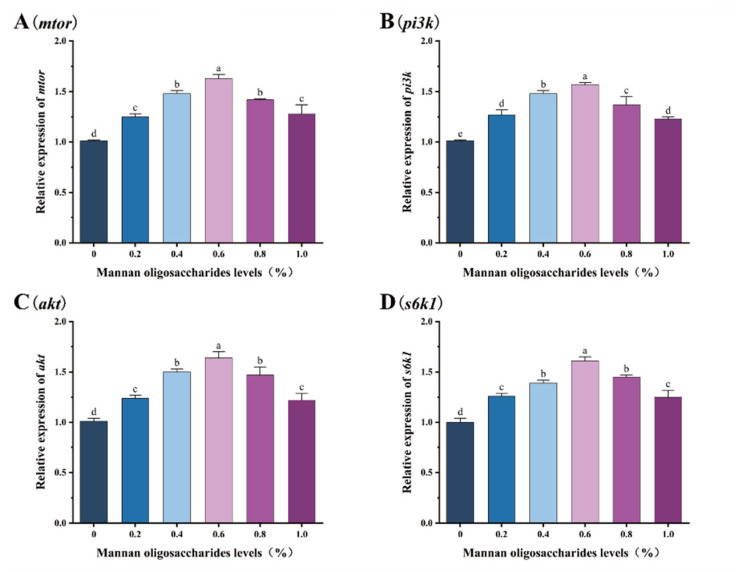
Effects of mannan oligosaccharide supplementation in feed on the relative expression of the mammalian target of rapamycin (*mtor*, (**A**)), phosphatidylinositol 3 kinase (*pi3k*, (**B**)), akt protein kinase B (*akt*, (**C**)), and ribosomal protein S6 kinase polypeptide 1 (*s6k1*, (**D**)) genes in the livers of juvenile GIFT tilapia. All of the above data are the mean ± SD (*n* = 3). Different superscript letters in the figure indicate significant differences among the data (*p* < 0.05).

The relative expression levels of the *mtor*, *pi3k*, *akt*, and *s6k1* genes in juvenile GIFT tilapia fed diets containing 0.20%, 0.40%, 0.60%, 0.80%, and 1.00% MOS were significantly higher than those in the control group (*p* < 0.05). Among these concentrations, the highest relative expression levels of *mtor*, *pi3k*, *akt*, and *s6k1* genes were observed in fish fed 0.60% MOS, and these levels were significantly higher than those in fish fed 0.20%, 0.40%, 0.80%, and 1.00% MOS (*p* < 0.05), as shown in Figure 6.

## 4. Discussion

### 4.1. MOS Supplementation Enhances Growth Performance

Growth performance indicators such as WGR, SGR, FCR, and PER are widely recognized as key parameters for evaluating the growth and feed utilization efficiency of aquatic animals. Typically, significant increases in WGR, SGR, and PER, accompanied by a marked reduction in FCR, are indicative of enhanced growth performance [28,29]. In the present study, juvenile GIFT tilapia fed diets supplemented with MOS exhibited significantly higher WGR, SGR, and PER, along with a significantly lower FCR, compared to the control group, with WGR, SGR, and PER increased by 44.53%, 15.17%, and 44.08%, respectively, and FCR reduced by 31.25%. The observed improvements may be partially attributed to the beneficial effects of MOS on intestinal morphology and microbial balance. MOS supplementation has been reported to increase intestinal villus height and muscularis thickness, thereby enlarging the nutrient absorption surface and promoting more efficient nutrient uptake. In addition, as a functional prebiotic, MOS can selectively stimulate the proliferation of beneficial gut microbes such as *Lactobacillus* and *Bifidobacterium*, while suppressing the growth of pathogenic bacteria. These changes contribute to a more favorable intestinal environment and enhanced digestive enzyme activity, ultimately improving feed utilization [30]. Moreover, MOS may play a role in supporting liver health by mitigating hepatic stress and reducing aminotransferase activity, allowing nutrients to be more effectively directed toward tissue synthesis and somatic growth [11]. Similar findings have been reported in other aquatic species such as pompano (*Trachinotus ovatus*) [31] and Nile tilapia [32], where dietary MOS supplementation significantly enhanced growth-related parameters (WGR, SGR, and PER) while simultaneously reducing FCR.

### 4.2. MOS Supplementation Affects Serum Biochemical Indices

Serum biochemical indicators are key parameters that reflect the health of fish. Changes in these indicators directly reflect feed nutrient utilization and metabolic function [33,34]. Serum TP and ALB levels are considered to be fundamental indicators for evaluating protein metabolism and immune status. Elevated levels have been shown to indicate improved protein synthesis efficiency and amino acid utilization, as well as improved nonspecific immune function [35]. The study demonstrated that the TP and ALB levels in the sera of juvenile GIFT tilapia in the MOS-added group were significantly higher, suggesting that MOS, as an active component of yeast cell walls, may contain polypeptides and active proteins that could directly promote the digestion and absorption of protein in the intestine, thereby increasing TP and ALB levels [36]. Moreover, the increase in ALB levels, together with the unchanged GLB levels, further supports the notion that the rise in TP levels is more likely attributable to the effect of MOS on nutrient absorption in juvenile tilapia, rather than on immune responses. These results are consistent with those of studies showing that administering 1.0–2.0 g/kg MOS to red sea bream (*Pagrus major*) feed significantly increased serum protein levels [37], and that administering 0.5–2.0% MOS to tilapia feed significantly increased the total protein content in serum [36]. LYZ, ACP, and ALP are critical components of the fish immune system, functioning as essential defenses against potentially harmful substances that could invade the organism. Elevated levels have been shown to indicate enhanced innate immune capacity, thereby strengthening disease resistance and reducing bacterial transport and survival [38]. The primary function of GLB is to serve as an indicator of liver function and the body’s immune status. Variations in GLB levels are context-dependent: elevated concentrations can be associated with liver dysfunction or bacterial infection, whereas reduced levels may be linked to impaired immune function and lower disease resistance [39]. In the present study, the serum levels of LYZ, ACP, and ALP in juvenile GIFT tilapia were significantly elevated in the MOS-supplemented group, while the GLB level showed no statistically significant difference compared with the control group. This pattern may reflect an adaptive immune modulation induced by MOS, potentially through the stimulation of liver secretion of mannose-binding lectin (MBL), which, in turn, activates the complement pathway, promotes immune responsiveness, and facilitates antigen processing. Correlative studies in Japanese eel (*Anguilla japonica*) have demonstrated that the administration of 5 g/kg MOS enhances lysozyme activity and intestinal morphology [40]. In the domain of liver function indicators, fluctuations in serum ALT, AST, and LDH levels frequently mirror the physiological condition of hepatopancreas cells, thereby providing a quantitative assessment of an organism’s stress level. A decline in these levels signifies an enhancement in the hepatopancreas’s physiological state, accompanied by a diminution in stress-induced damage [41]. The present study demonstrated that the serum levels of ALT, AST, and LDH in juvenile GIFT tilapia were significantly reduced in the MOS-added group. This phenomenon can be attributed to the reduction in pathogenic microorganisms in the intestine and the decrease in the production of harmful metabolites by MOS as a prebiotic. Thus, MOS alleviates stress damage to the hepatopancreas [42]. This finding is consistent with the results of a study on carp (*Cyprinus carpio*), which demonstrated that MOS reduced serum ALT and AST levels while increasing total protein (TP) and albumin (ALB) levels [11]. In the domain of lipid metabolism, triglycerides serve as a critical indicator of the body’s metabolic status [43]. The HDL/LDL ratio has been demonstrated to reflect the transport status of cholesterol in fish and serves as an indicator of the severity of atherosclerosis. An elevated ratio signifies that cholesterol is being transported more expeditiously within the body, thereby reducing its storage and maintaining overall health [44]. The present study demonstrated that the sera of juvenile GIFT tilapia in the MOS group exhibited a significant increase in HDL content, accompanied by a significant decrease in TG and LDL contents. These observations may be attributed to the enhancement of the intestinal microbiota structure of the host and the increase in short-chain fatty acids (SCFAs) produced by MOS [45]. SCFAs have been identified as stimulators of fatty acid oxidation, a process that facilitates the entry of fatty acids into mitochondria for the subsequent β-oxidation reaction. This activation is achieved through the stimulation of the peroxisome proliferator-activated receptor (PPAR) pathway, a crucial regulatory mechanism in metabolic processes. The net effect of this stimulation is a reduction in serum TG levels, accompanied by an enhancement of the HDL/LDL ratio [46]. A similar study was conducted on gilthead sea bream (*Sparus aurata*), which demonstrated that MOS significantly increased the ALP content and reduced the triglyceride content of the fish [47]. Serum glucose levels are influenced by nutritional intake and metabolism. The monitoring of changes in glucose levels can provide insights into energy balance and nutrient absorption in animal organisms [48]. The present study demonstrated that the GLU content in the sera of juvenile GIFT tilapia in the MOS-added group exhibited a significant increase. This phenomenon may be attributed to the observed improvement in intestinal function and the impact on intestinal microbiota by MOS, consequently affecting the absorption of nutrients in feed [49]. In addition, MOS enhanced the activity of α-amylase, which resulted in an increased starch decomposition rate and, consequently, an elevated serum glucose level [50]. A similar body of research has been conducted on largemouth bass, with the addition of 5 g/kg MOS to their feed resulting in a significant increase in serum glucose levels [51].

### 4.3. MOS Supplementation Affects Muscle Composition

Muscle composition is regarded as a reliable analytical factor for evaluating the nutritional value, physiological status, and habitat of fish [52]. Determining the contents of crude protein, moisture, crude lipid, and crude ash in fish muscle is crucial for understanding the nutritional status of fish, as these parameters serve as important indicators of physiological condition and product quality [53]. In the present study, no statistically significant differences were observed in crude protein, moisture, or crude ash contents between the MOS-supplemented groups and the control group. The moisture and ash contents in the muscle tissue of fish fed MOS-containing diets were comparable to those in the control group, suggesting that dietary MOS supplementation did not markedly influence these parameters. However, the crude lipid content in the muscle of fish in the 0.60% MOS group was significantly higher than that in the control group. This outcome may be attributable to the direct interaction of MOS with intestinal epithelial cells, resulting in an elevated nutrient exchange rate, thereby enhancing nutrient digestibility, feed utilization, and intestinal lipid absorption [5]. Crude ash and moisture content are mainly affected by the mineral content in the body. The dynamic stability of mineral content can maintain water and salt balance in fish [54,55]. The crude ash and moisture contents of juvenile GIFT tilapia showed no significant differences among the treatment groups, which may be explained by the fact that dietary MOS did not affect mineral absorption. Crude protein content is primarily influenced by the protein level in the diet [56]. Although MOS supplementation affected protein absorption in juvenile GIFT tilapia, the inclusion level was insufficient to produce a significant difference in crude protein content among groups. Similar findings were reported in juvenile giant sturgeon (*Huso huso*), where dietary MOS supplementation increased the crude lipid content but did not alter the crude ash, crude protein, or moisture contents [57]. The absence of significant changes in most muscle composition parameters in the present study may also be due to the relatively short rearing period, which may have been insufficient to induce tissue-level alterations. Instead, MOS is more likely to enhance nutrient absorption and promote physiological functions, rather than directly altering muscle composition [58].

### 4.4. MOS Supplementation Enhances Digestive Enzyme Activity

In aquatic animals, intestinal digestive enzyme activity serves as a critical indicator of the efficiency of nutrient digestion and absorption in fish. Among these enzymes, trypsin, α-amylase, and lipase are closely related to the digestion of proteins, sugars, and lipids [59,60]. The experiment demonstrated that the activity of lipase, α-amylase, and trypsin in the foreguts of fish in the MOS group was considerably higher than that in the control group. This finding suggests that MOS could enhance the activity of digestive enzymes in the intestine, thereby promoting the absorption and utilization of nutrients by fish. The potential mechanism underlying this effect might involve MOS’s impact on the diversity of intestinal microorganisms and the improvement of the intestinal absorption area [61]. Specifically, MOS has been shown to significantly increase the length and density of intestinal microvilli, thus enhancing the contact efficiency between digestive enzymes and substrates. In contrast, the administration of MOS has been shown to promote the proliferation of beneficial bacteria, such as *Lactobacillus* and *Bifidobacteria*, within the gastrointestinal tract. The metabolites of beneficial bacteria have been shown to promote the shedding of small intestinal epithelial cells, thereby facilitating the entry of digestive enzymes secreted by epithelial cells into the digestive tract. In addition to its primary function, it has been observed to enhance intestinal peristalsis and increase digestive enzyme activity [62]. A series of studies conducted on largemouth bass (*Micropterus salmoides*) [63], juvenile narrow-clawed crayfish (*Astacus leptodactylus*) [64], juvenile striped catfish (*Pangasianodon hypophthalmus*) [65], and yellow catfish (*Pelteobagrus fulvidraco*) [66] have demonstrated that the addition of MOS to feed significantly increases digestive enzyme activity. Meanwhile, Pearson correlation analysis between trypsin, α-amylase, and lipase activities and WGR, SGR, and PER revealed significant positive correlations (r ≈ 0.7, *p* < 0.05), further suggesting that MOS is most likely to enhance the growth performance of juvenile tilapia by improving the activities of these digestive enzymes.

### 4.5. MOS Supplementation Enhances Antioxidant and Immune Performance

The expression levels of antioxidant enzyme genes serve as critical indicators for evaluating the oxidative stress defense capacity of fish. Among these, the Nrf2 signaling pathway serves as a key regulatory hub for antioxidant defense in the body. It achieves this by regulating the transcriptional activity of downstream antioxidant enzymes (such as *sod*, *cat*, *gsh-px*, and *gst*) through the dynamic balance between Nrf2 and Keap1 [67,68]. In this experiment, the expression levels of the *sod*, *cat*, *gsh-px*, *gst*, and *nrf2* genes in the livers of fish in the MOS group were found to be significantly higher than those in the control group. This finding suggests that MOS can significantly boost the expression levels of antioxidant enzyme genes, thereby increasing the antioxidant capacity of fish. The underlying reason for this phenomenon could be attributed to the presence of hydroxyl functional groups in MOS, which possess the capacity to eliminate ROS in fish. This elimination was facilitated through electron transfer, a process that also results in the removal of free radicals. Consequently, this process led to a reduction in oxidative damage and an upregulation in the expression of antioxidant genes [69,70]. Additionally, MOS has been observed to facilitate the activation of the antioxidant signaling molecule Nrf2 within the body. This process involves the nuclear translocation of Nrf2, where it subsequently binds to antioxidant response elements located within the cell nucleus. This binding event serves to initiate the transcription of antioxidant enzyme genes within the downstream pathway, thereby contributing to the overall antioxidant response [71]. In this study, the upregulation of antioxidant-related genes such as sod, cat, and nrf2 was accompanied by a reduction in serum liver damage markers, including ALT, and AST, and no significant accumulation of lipid peroxidation products (e.g., MDA) was observed. These findings suggest that the antioxidant activation induced by MOS is a preventive mechanism rather than a passive response following oxidative damage. Nrf2 serves as a central transcription factor in antioxidant defense; its upregulation can actively trigger the synthesis of downstream antioxidant enzymes. This represents an adaptive physiological response to prebiotic intervention, which fundamentally differs from the stress-induced activation observed under pathological conditions, typically associated with tissue injury [66,70]. Similar studies have been conducted on grass carp, where the addition of 400–600 mg/kg MOS to the diet significantly increased the expression of genes such as *nrf2*, *sod*, and *cat*, thereby enhancing the fish’s antioxidant capacity [71]. In a study on Nile tilapia, the addition of BIO-MOS^®^ (Alltech Inc., Nicholasville, KY, USA) to the diet significantly increased the mRNA expression of *gsh-px* and *sod* [72].

Nonspecific immunity plays a crucial role in the immune systems of fish. Substances such as lysozyme, alkaline phosphatase, and cytokines recognize and eliminate various pathogens and foreign substances. They also resist the invasion of harmful substances from the external environment. These substances are important components of the nonspecific immune system [38]. LYZ and ALP are critical innate immune factors capable of killing bacteria and impeding bacterial infections [73,74]. Lysozyme possesses the ability to directly lyse bacterial cell walls, and its upregulation can enhance defense against Gram-positive bacterial invasion, thereby strengthening innate immunity [75]. The expression levels of the *lyz* and *alp* genes are positively correlated with their enzyme activity and enzyme content [76]. The present study demonstrated that the expression levels of *lyz* and *alp* genes in the livers, head kidneys, and spleens of fish in the MOS group were significantly higher than those in the control group. This phenomenon may be attributed to the ability of MOS to activate macrophages, augment their population, and stimulate the secretion of cytokines and lysozyme [77]. A similar research approach has been employed in the context of juvenile Jian carp, wherein MOS was utilized to substitute for a portion of the fish meal in the fish feed. This resulted in an elevated expression of the *alp* gene [78]. Similarly, in a study on zebrafish, feeding diets containing different levels of Agrimos^®^ MOS (Lallemand Animal Nutrition, Blagnac, France) (MOS, 0.2%, 0.4%, and 0.8%) for 90 days resulted in a significant upregulation of the *lyz* gene in the intestines of adult fish and their offspring, with the most pronounced effect observed in the 0.4–0.8% groups, accompanied by a decrease in Lyz locus methylation [79].

Heat shock proteins (HSPs) are molecular chaperones that play a crucial role in maintaining protein homeostasis, facilitating the repair of cellular damage, and enhancing tolerance to diverse stressors. The expression of HSP genes, particularly *hsp70*, can be triggered by a variety of stimuli, including environmental challenges, dietary interventions, and changes in intestinal microbiota composition, rather than being limited to heat shock alone [80]. In the present study, *hsp70* expression in the livers, head kidneys, and spleens of MOS-fed fish was significantly higher than in the control group. This upregulation is likely linked to the ability of MOS to promote the proliferation of beneficial intestinal bacteria, which, in turn, can modulate host immune responses and activate cytoprotective pathways, including HSP-mediated stress protection [13]. Regarding the *hsp* gene, its increased expression coincided with the downregulation of pro-inflammatory cytokines, such as *tnf-α* and *il-1β*. This, together with the observed proliferation of beneficial gut microbiota (e.g., *Lactobacillus*), indicates that hsp is primarily involved in maintaining cellular homeostasis and modulating immune responses, rather than solely functioning as a stress-response protein [12,78]. Comparable results have been observed in allogynogenetic crucian carp (*Carassius auratus gibelio*), where dietary supplementation with 480 mg/kg MOS led to a marked increase in hepatic *hsp70* mRNA expression compared with the control [81].

Inflammatory cytokines are typically classified into pro-inflammatory cytokines (TNF-α, IL-1β, IL-6, IL-8, INF-γ) and anti-inflammatory cytokines (IL-10, TGF-β), based on their response effect types. Downregulating the mRNA levels of pro-inflammatory cytokines and upregulating the mRNA levels of anti-inflammatory cytokines can mitigate excessive inflammatory responses [82]. The present study demonstrated that the expression levels of the *tnf-α*, *il-1β*, il-6, *il-8*, and *inf-γ* genes in the livers, head kidneys, and spleens of fish in the MOS group were significantly lower than those in the control group. Conversely, the expression levels of the *il-10* and *tgf-β* genes were significantly higher in the MOS group than in the control group. The mechanism may be that MOS acts as an exogenous antigen adjuvant, regulating T-cell immune responses, enhancing immune function, and inhibiting excessive inflammatory responses [83,84]. A similar body of research has demonstrated that the incorporation of MOS into the diets of juvenile hybrid groupers (*Epinephelus lanceolatus ♂* × *Epinephelus fuscoguttatus ♀*) has a substantial impact on the expression level of the *tgf-β1* gene [85]. Furthermore, the addition of 2000 mg/kg MOS to the diet of turbot (*Scophthalmus maximus*) has been shown to result in a notable increase in the mRNA levels of *tgf-β* within the intestines of the fish [86].

### 4.6. MOS Supplementation Affects mTOR Signaling Pathway

The mTOR pathway has been confirmed to regulate cellular metabolism, growth, proliferation, and survival. Activation of the pathway occurs during various cellular activities, with signaling proteins combining to affect the immune system, antioxidant function, growth, and other processes [17]. The mTOR signaling pathway primarily regulates cell growth, proliferation, and autophagy by acting on PI3K and AKT as upstream regulators and on 4E-BP1 and S6K1 as downstream regulators. Upregulation of the *mtor*, *s6k1*, *pi3k*, and *akt* genes activates the mTOR signaling pathway, increasing cell growth and proliferation while reducing cell autophagy [87]. This experiment showed that adding MOS to the feed significantly increased the expression of the *mtor*, *s6k1*, *pi3k*, and *akt* genes. This may be because MOS increases *Lactobacillus* content in fish intestines, and Lactobacillus ferments and produces riboflavin [88]. Riboflavin, an essential micronutrient, influences the expression of the mTOR and S6K1 genes [89]. On the other hand, MOS may activate the extracellular signal-regulated kinase and AKT signaling pathways by binding to cell surface receptors, such as SIGNR1. This promotes the phosphorylation and activity of mTORC1, ultimately activating the mTOR pathway [90]. Similar studies have found that feeding grass carp (*Ctenopharyngodon idella*) MOS increases *s6k1* gene expression [91].

## 5. Conclusions

In conclusion, a comprehensive analysis of the experimental results demonstrated that supplementing the diets of juvenile GIFT tilapia with different levels of MOS significantly affected various physiological parameters, such as growth performance, serum biochemical profiles, muscle composition, digestive enzyme activities, antioxidant and immune functions, as well as the mTOR signaling pathway. The study revealed that 0.60% MOS supplementation yielded the most favorable outcomes. Notably, the activation of metabolism- and growth-related pathways, along with enhanced antioxidant and immune responses observed in this study, is more likely to represent a positive adaptive adjustment of the organism to dietary MOS intervention rather than a pathological stress response. This adjustment includes improved nutrient utilization efficiency, strengthened cellular defense systems, and optimized growth-regulatory signaling, reflecting a positive modulation of the overall physiological status of the fish. However, given the relatively short duration of the present feeding trial, further validations under different rearing conditions, extended feeding periods, and commercial-scale applications are warranted to confirm the stability, feasibility, and practical applicability of these findings.

## Figures and Tables

**Table 1:** Composition of the experimental diets for juvenile tilapia (g/kg of dried feed).

Ingredients	Mannan Oligosaccharides Levels (%)					
	0	0.2	0.4	0.6	0.8	1.0
Mannan oligosaccharides	0.00	2.00	4.00	6.00	8.00	10.00
Soybean oil	30.00	30.00	30.00	30.00	30.00	30.00
Fish oil	10.00	10.00	10.00	10.00	10.00	10.00
Chicken meal	80.00	80.00	80.00	80.00	80.00	80.00
Rapeseed meal	200.00	200.00	200.00	200.00	200.00	200.00
Fermented soybean meal	350.00	350.00	350.00	350.00	350.00	350.00
Dextrin	243.90	241.90	239.90	237.90	235.90	233.90
Gelatin	50.00	50.00	50.00	50.00	50.00	50.00
Vitamins mixture *	10.00	10.00	10.00	10.00	10.00	10.00
Minerals mixture **	10.00	10.00	10.00	10.00	10.00	10.00
Choline chloride	5.00	5.00	5.00	5.00	5.00	5.00
Sodium chloride	5.00	5.00	5.00	5.00	5.00	5.00
Adhesive	5.00	5.00	5.00	5.00	5.00	5.00
Attractant	0.10	0.10	0.10	0.10	0.10	0.10
Preservative	1.00	1.00	1.00	1.00	1.00	1.00
Proximate composition (%)						
Crude protein	34.40	34.40	34.40	34.40	34.40	34.40
Crude lipid	7.88	7.88	7.88	7.88	7.88	7.88
Ash	7.66	7.66	7.66	7.66	7.66	7.66
Moisture	9.20	9.20	9.20	9.20	9.20	9.20
Crude fiber	5.26	5.26	5.26	5.26	5.26	5.26
Gross energy (Mcal/kg)	3.90	3.90	3.90	3.90	3.90	3.90

* Vitamins mixture (IU or mg/kg of dried feed): vitamin A 2500 IU; vitamin D_3_ 1200 IU; vitamin K_3_ 60 IU; folic acid 5 mg; vitamin B_1_ 10 mg; vitamin B_2_ 10 mg; vitamin B_6_ 20 mg; vitamin B_12_ 0.15 mg; niacin 40 mg; calcium pantothenate 20 mg; inositol 150 mg; biotin 0.2 mg; vitamin C 150 mg; vitamin E 60 mg. ** Minerals mixture (mg/kg of dried feed): iron 15 mg; zinc 20 mg; manganese 2 mg; copper 1 mg; iodine 0.2 mg; selenium 0.05 mg; cobalt 0.25 mg; magnesium 0.06 mg; potassium 40 mg.

**Table 2:** The primer sequence of detected genes for RT-qPCR.

Gene	Primer Sequence (5′-3′)	Tm (°C)	Product Size (bp)	GenBank	R^2^	Slope	Efficiency (%)
*β-actin* ^1^	F: AAGGACCTGTACGCCAACAC	60	196	KJ126772.1	0.991	−3.30	100.92
	R: ACATCTGCTGGAAGGTGGAC						
*sod* ^2^	F: GTCTGCTGTTACGGTGGCTGTAC	60	82	XM_003449940.5	0.995	−3.40	96.84
	R: ATCAATGCGAAGTCTTCCACTGTCC						
*cat* ^3^	F: TTGAAGGCTGTGCATCCAGACTATG	60	129	XM_003447521.5	0.993	−3.35	98.84
	R: TGAGGCGGTGATGGCTGAGG						
*gsh-px* ^4^	F: AAAATGTGGCGTCTCTCTGAGGAAC	60	85	NM_001279711.1	0.997	−3.29	101.35
	R: AGACCTTCGGCGGAGTAGCG						
*gst* ^5^	F: TTGCTGATGTGCTGCTTGTTGAATG	60	125	XM_025897213.1	0.992	−3.41	96.45
	R: CCTGCTGATGGCGGGGATTTG						
*nrf2* ^6^	F: GGAAATGAAAGTGCTGCTGTGTC	60	134	XM_003447296.5	0.993	−3.24	103.54
	R: TCTGAGTCTGGCTGTTCTGTTATTAG						
*lyz* ^7^	F: GCCGCTGGTGGTGCAATGAC	60	148	XM_013265574.3	0.998	−3.29	101.35
	R: CAGGCAACCCAGGCTGTGATG						
*alp* ^8^	F: ATGGAGGAGAGGATGTGGCTGTG	60	128	XM_005469634.4	0.992	−3.45	94.92
	R: GTGTTCCCTGTTCTGCCCGATAC						
*tnf-α* ^9^	F: TCGTCGTCGTGGCTCTTTGTTTAG	60	100	NM_001279533.1	0.991	−3.38	97.63
	R: AGTGCTTCTGGCTGTCCTAATTGTG						
*il-1β* ^10^	F: ACAAGGATGACGACAAGCCAACC	60	147	XM_019365844.2	0.996	−3.36	98.43
	R: GGACAGACATGAGAGTGCTGATGC						
*il-6* ^11^	F: GATGCTGGCCGCTCTGCTTC	60	100	XM_019350387.2	0.994	−3.37	98.03
	R: CATCTCCGCCTCCTCTGTCICC						
*il-8* ^12^	F: CTGTGAAGGCATGGGTGTGGAG	60	136	NM_001279704.1	0.997	−3.33	99.66
	R: CAGTGTGGCAATGATCTCTGTCTCC						
*il-10* ^13^	F: TGGAGAGCAGAGGTCTATACAAGGC	60	117	XM_013269189.3	0.992	−3.32	100.09
	R: TCAGCAGGTCTTCGAGCAGAGG						
*inf-γ* ^14^	F: GAAACAACTGCCCACTCCGAGTC	60	110	NM_001287402.1	0.994	−3.35	98.84
	R: TGCCTGGTAGCGAGCCTGAG						
*tgf-β* ^15^	F: TGCCTCCTCTCCACTGAGTGATTC	60	80	NM_001311325.1	0.990	−3.44	95.30
	R: CTCCTCCGACTTCCCTTTCAATGC						
*hsp70* ^16^	F: CAAGGTGATTTCAGACGGAGGGAAG	60	123	XM_003442456.5	0.995	−3.43	95.68
	R: GCCTCTGCGATCTCCTTCATCTTC						
*mtor* ^17^	F: TGACCATCCTCAACCTGCTTCC	60	123	XM_003449131.5	0.992	−3.40	96.84
	R: CCGTCCTCTCCTTCTCCTTCTTC						
*akt* ^18^	F: ATGATGTGCGGTAGACTGCCTTTC	60	130	XM 003447818.5	0.997	−3.38	97.63
	R: TCAGAAGACCAGAGAGCAGAGAGC						
*s6k1* ^19^	F: CGGTGTCCTCCAGTCTCCTC	60	150	NM_001287402.1	0.992	−3.42	96.06
	R: GGATAGGCTTGCTGCTTCATCTG						
*pi3k* ^20^	F: GATGAAGAGGCGTCGGTGTGAAC	60	110	XM_005463451.4	0.993	−3.38	101.78
	R: AGAGCGGCGAAGTCCAGGATG						

Note: F: forward primer; R: reverse primer. ^1^ *β-actin*: the non-regulatory internal reference gene; ^2^ *sod*: superoxide dismutase; ^3^ *cat*: catalase; ^4^ *gsh-px*: glutathione peroxidase; ^5^ *gst*: glutathione S-transferase; ^6^ *nrf2*: nuclear factor erythroid 2-related factor 2; ^7^ *lyz*: lysozyme; ^8^ *alp*: alkaline phosphatase; ^9^ *tnf-α*: tumor necrosis factor α; ^10^ *il-1β*: interleukin-1 beta; ^11^ *il-6*: interleukin-6; ^12^ *il-8*: interleukin-8; ^13^ *il-10*: interleukin-10; ^14^ *inf-γ*: interferon gamma; ^15^ *tgf-β*: transforming growth factor β; ^16^ *hsp70*: heat shock protein 70; ^17^ *mtor*: mammalian target of rapamycin; ^18^ *akt*: Akt protein kinase B; ^19^ *pi3k*: phosphatidylinositol 3 kinase; ^20^ *s6k1*: ribosomal protein S6 kinase polypeptide 1.

**Table 3:** Effects of mannan oligosaccharide supplementation in feed on serum biochemical parameters of the juvenile GIFT tilapia.

Index	Mannan Oligosaccharide Levels (%)					
	0	0.20	0.40	0.60	0.80	1.00
TP ^1^ (g/L)	11.22 ± 1.49 ^c^	12.64 ± 0.78 ^bc^	15.03 ± 1.94 ^ab^	15.29 ± 0.85 ^ab^	15.77 ± 1.32 ^a^	15.73 ± 2.01 ^a^
ALB ^2^ (g/L)	6.08 ± 0.19 ^c^	7.59 ± 0.12 ^b^	8.19 ± 0.19 ^b^	8.09 ± 0.21 ^b^	7.93 ± 0.35 ^b^	9.56 ± 0.85 ^a^
GLB ^3^ (g/L)	5.14 ± 1.39 ^a^	5.72 ± 0.43 ^a^	6.64 ± 1.80 ^a^	5.73 ± 1.68 ^a^	7.69 ± 1.32 ^a^	7.80 ± 2.27 ^a^
TG ^4^ (mmol/L)	0.68 ± 0.03 ^a^	0.55 ± 0.04 ^bc^	0.50 ± 0.04 ^c^	0.23 ± 0.01 ^e^	0.41 ± 0.01 ^d^	0.57 ± 0.03 ^b^
GLU ^5^ (mmol/L)	2.71 ± 0.55 ^c^	3.83 ± 0.59 ^bc^	4.47 ± 0.64 ^ab^	4.79 ± 0.52 ^ab^	4.96 ± 0.62 ^ab^	5.18 ± 0.83 ^a^
HDL ^6^ (mmol/L)	0.57 ± 0.09 ^d^	0.76 ± 0.08 ^c^	1.01 ± 0.08 ^b^	1.31 ± 0.05 ^a^	0.95 ± 0.05 ^b^	0.79 ± 0.12 ^c^
LDL ^7^ (mmol/L)	2.28 ± 0.11 ^a^	1.82 ± 0.20 ^b^	1.63 ± 0.20 ^b^	1.11 ± 0.30 ^c^	1.56 ± 0.11 ^b^	1.76 ± 0.11 ^b^
AST ^8^ (U/L)	81.62 ± 1.47 ^a^	75.41 ± 1.02 ^b^	72.58 ± 1.87 ^bc^	70.20 ± 2.41 ^c^	71.75 ± 2.18 ^c^	71.72 ± 0.92 ^c^
ALT ^9^ (U/L)	72.72 ± 0.97 ^a^	67.60 ± 1.09 ^b^	63.62 ± 1.71 ^c^	62.70 ± 1.75 ^c^	63.60 ± 1.09 ^c^	63.09 ± 1.63 ^c^
LYZ ^10^ (μg/mL)	2.66 ± 0.15 ^e^	3.60 ± 0.15 ^c^	4.16 ± 0.10 ^b^	4.87 ± 0.15 ^a^	4.09 ± 0.12 ^b^	3.25 ± 0.34 ^d^
ALP ^11^ (King Unit/100 mL)	6.08 ± 0.28 ^b^	6.69 ± 0.14 ^ab^	7.21 ± 0.33 ^a^	7.26 ± 0.38 ^a^	7.30 ± 0.62 ^a^	7.24 ± 0.26 ^a^
ACP ^12^ (King Unit/100 mL)	6.02 ± 0.35 ^b^	6.49 ± 0.31 ^ab^	7.15 ± 0.64 ^ab^	7.31 ± 0.38 ^a^	7.68 ± 1.21 ^a^	7.51 ± 0.26 ^a^
LDH ^13^ (U/L)	1038.46 ± 140.85 ^a^	945.59 ± 66.77 ^ab^	825.92 ± 96.30 ^b^	807.19 ± 104.13 ^b^	793.27 ± 84.85 ^b^	844.78 ± 82.03 ^b^

Notes: All od the above data are the mean ± SD (*n* = 3). Different superscript letters in the same row indicate significant differences among the data (*p* < 0.05).^1^ TP: total protein; ^2^ ALB: albumin; ^3^ GLB: globulin; ^4^ TG: triglyceride; ^5^ GLU: glucose; ^6^ HDL: high-density lipoprotein; ^7^ LDL: low-density lipoprotein; ^8^ AST: aspartate transaminase; ^9^ ALT: alanine transaminase; ^10^ LYZ: lysozyme; ^11^ ALP: alkaline phosphatase; ^12^ ACP: acid phosphatase; ^13^ LDH: lactate dehydrogenase.

**Table 4:** Effects of mannan oligosaccharide supplementation in feed on the muscle composition of juvenile GIFT tilapia.

Index	Mannan Oligosaccharide Levels (%)					
	0	0.20	0.40	0.60	0.80	1.00
Crude protein (%)	17.85 ± 1.23	17.44 ± 1.25	17.59 ± 0.26	18.52 ± 0.89	18.08 ± 2.30	18.37 ± 0.77
Crude lipid (%)	1.47 ± 0.11 ^b^	1.62 ± 0.13 ^b^	1.65 ± 0.20 ^b^	1.88 ± 0.42 ^a^	1.54 ± 0.17 ^b^	1.45 ± 0.07 ^b^
Ash (%)	1.42 ± 0.23	1.40 ± 0.14	1.42 ± 0.16	1.38 ± 0.13	1.41 ± 0.04	1.44 ± 0.12
Moisture (%)	79.48 ± 0.28	79.58 ± 1.27	79.39 ± 1.12	78.18 ± 0.44	79.20 ± 2.72	79.05 ± 1.54

Notes: All of the above data are the mean ± SD (*n* = 3). Different superscript letters in the same row indicate significant differences among the data (*p* < 0.05).

**Table 5:** Effects of mannan oligosaccharide supplementation in feed on the digestive enzyme activity of juvenile GIFT tilapia.

Index	Mannan Oligosaccharide Levels (%)					
	0	0.20	0.40	0.60	0.80	1.00
Lipase (U/mg prot)	683.22 ± 26.96 ^d^	732.72 ± 10.06 ^bc^	774.01 ± 17.09 ^b^	827.88 ± 33.56 ^a^	740.61 ± 11.89 ^bc^	727.15 ± 29.30 ^c^
α-Amylase (U/mg prot)	257.91 ± 48.27 ^c^	317.32 ± 5.22 ^b^	347.33 ± 6.43 ^b^	442.85 ± 16.78 ^a^	352.86 ± 4.70 ^b^	324.08 ± 36.19 ^b^
Trypsin (U/mg prot)	1580.82 ± 53.95 ^c^	1642.47 ± 13.84 ^b^	1667.67 ± 23.37 ^b^	1747.49 ± 21.68 ^a^	1652.49 ± 19.56 ^b^	1633.07 ± 18.84 ^b^

Note: All of the above data are the mean ± SD (*n* = 3). Different superscript letters in the same row indicate significant differences among the data (*p* < 0.05).

## Data Availability

The data presented in this study are available upon request from the first author.
